# Perceived Information Overload and Unverified Information Sharing on WeChat Amid the COVID-19 Pandemic: A Moderated Mediation Model of Anxiety and Perceived Herd

**DOI:** 10.3389/fpsyg.2022.837820

**Published:** 2022-02-03

**Authors:** Qing Huang, Sihan Lei, Binbin Ni

**Affiliations:** ^1^College of Media and International Culture, Zhejiang University, Hangzhou, China; ^2^School of Communication, East China Normal University, Shanghai, China

**Keywords:** unverified information sharing, perceived information overload, anxiety, perceived herd, moderated mediation, COVID-19

## Abstract

Individuals’ unverified information sharing on social media, namely, sharing information without verification, is a major cause of the widespread misinformation amid the COVID-19 pandemic. The association between perceived information overload and unverified information sharing has been well documented in the cognitive overload approach. However, little is known about the underlying mechanism of this process. This study aims to explore the mediating role of anxiety and the moderating role of perceived herd between perceived information overload and unverified information sharing on WeChat. Anxiety demonstrates people’s emotional response to the pandemic, whereas perceived herd describes a willingness to share certain information if it has been shared by many. The results of an online survey in China (*N* = 525) showed that perceived information overload was positively associated with unverified information sharing. In addition, this relationship was partially mediated by anxiety. Moreover, perceived herd positively moderated the link between anxiety and unverified information sharing, such that the indirect effect of perceived information overload on unverified information sharing *via* anxiety was significant in conditions where the level of perceived herd was high, whereas the indirect effect was not significant in conditions where the level of perceived herd was low. The moderated mediation model extends the cognitive overload approach and indicates that unverified information sharing is not only an individual strategy to cope with information overload but also a herding behavior to manage anxiety. Practical implications for curbing people’s tendencies toward unverified information sharing on social media are discussed.

## Introduction

The COVID-19 pandemic has not only posed a severe threat to public health but has also brought about an infodemic. An infodemic occurs when an excessive amount of information, including false or misleading information, circulates in digital and physical environments during a disease outbreak, which leads to public confusion, risk-taking behaviors, mistrust in health authorities, and other negative social impacts (WHO, 2020a). The prevalence of social media amplifies this phenomenon because information travels much faster and further compared with the times when web-based technologies are not prevalent (Zarocostas, 2020). Moreover, social media afford users the great convenience of sharing information with just a click, usually without careful scrutiny of the information content (Apuke and Omar, 2021). The individual-level behavior of sharing information without verification is a major cause of the wide spread of misinformation. Thus, understanding individuals’ unverified information sharing on social media is of great importance in fighting the infodemic.

In relation to the term *unverified information sharing*, several other terms, such as *misinformation sharing* and *fake news sharing*, have been used interchangeably in extant research (Islam et al., 2020; Laato et al., 2020; Apuke and Omar, 2021). However, we suggest important differences between them. Unverified information sharing emphasizes people’s sharing without authenticating the information (Laato et al., 2020), and the shared information could be either true or false. By contrast, misinformation sharing refers to people’s sharing of incorrect information that is created without the intention of causing harm (Madraki et al., 2021), whereas fake news sharing describes individuals’ sharing of false information that is intentionally created to mislead readers (Di Domenico et al., 2021). The rapidly evolving situation of the COVID-19 pandemic and the information overload have made it increasingly difficult for ordinary people to differentiate between misinformation, fake news, and facts (Eysenbach, 2020; Huynh, 2020). In most cases, individuals do not intentionally share misinformation or fake news when they realize the information is incorrect (Mena, 2020). Nevertheless, not knowing the veracity of information and sharing it without verification is quite common (Islam et al., 2020; Laato et al., 2020). Thus, we consider unverified information sharing an appropriate term.

A majority of prior research has used a psychological perspective to explicate unverified information sharing. The first research line has adopted the uses and gratifications theory and viewed unverified information sharing as a behavior motivated by fulfilling certain needs, such as socialization, self-promotion, pass time, entertainment, and altruism (Islam et al., 2020; Apuke and Omar, 2021; Balakrishnan et al., 2021). The second line has employed the cognitive overload approach and assumed that human brains overloaded by information have limited processing capability; to cope with cognitive overload, people tend to share information without authentication (Fox et al., 2007; Talwar et al., 2019; Laato et al., 2020). In relation to unverified information sharing, the third line has identified that negative emotions, especially anxiety, are a significant predictor of people’s information-sharing behaviors (Rosnow, 1991; He et al., 2019; Lim et al., 2021).

Although the uses and gratifications theory has illuminated the motives of people’s information sharing on social media, such as socialization, self-promotion, entertainment, pass time, and altruism (Islam et al., 2020; Apuke and Omar, 2021; Balakrishnan et al., 2021), these motives do not capture the uniqueness of sharing without verification. In other words, people share information without verifying its content, mainly because they have limited processing capability when faced with the uncertainty of the pandemic and the excessive amount of rapidly updating information (Fox et al., 2007; Sweller, 2011). Thus, we propose that the cognitive overload approach is more appropriate than the uses and gratifications theory to explain unverified information sharing in this study. Furthermore, a plethora of research has shown that repeated and excessive exposure to COVID-19 information can potentially induce anxiety and other related negative emotions (Bao et al., 2020; Nekliudov et al., 2020; Zou et al., 2021), which suggests that the cognitive overload approach and the emotional predictors should be integrated to understand unverified information sharing. Moreover, unverified information sharing is not only an individual behavior to cope with information overload and the associated anxiety (He et al., 2019; Talwar et al., 2019; Laato et al., 2020; Lim et al., 2021) but is also susceptible to others’ influence, especially on social media (Apuke and Omar, 2020). Thus, social influence should be considered when examining unverified information sharing on social media.

Based on the cognitive overload approach (Fox et al., 2007; Samson and Kostyszyn, 2015; Laato et al., 2020; Whelan et al., 2020), this study introduces perceived information overload as a predictor of unverified information sharing. Furthermore, given that cognitive overload is often associated with negative emotions, especially anxiety (Bao et al., 2020; Nekliudov et al., 2020; Zou et al., 2021), we treat anxiety as a mediator between perceived information overload and unverified information sharing. According to the social impact theory (Latané, 1981; Handarkho, 2020), social media create situations in which individuals can observe others’ behaviors, which generates pressure for individual users to follow the crowd. Thus, we include perceived herd, a willingness to share a piece of information when shared by many on social media (Apuke and Omar, 2020), as a moderator in the mediating relationship. In particular, WeChat is the most widely used smartphone application for people to acquire information or news about COVID-19 in China (Liu, 2020). A considerable amount of misinformation related to the COVID-19 pandemic has been circulating on WeChat (Naeem and Bhatti, 2020). Thus, we test the moderated mediation model of unverified information sharing on WeChat. The results would provide us with a comprehensive understanding of the socio-psychological mechanism of unverified information sharing on social media and offer new directions for curbing the widespread misinformation.

## Theoretical Background and Hypothesis Development

### Perceived Information Overload and Unverified Information Sharing

During the COVID-19 pandemic, a vast number of messages created by multiple sources, such as scientists, government and health agencies, news media, key opinion leaders, and ordinary social media users, have been widely circulating on various social media platforms worldwide (WHO, 2020b). This is also the case for WeChat in China (Ma et al., 2020). In the face of a huge amount of information, individuals tend to feel overwhelmed, which is termed perceived information overload in prior research (Hong and Kim, 2020). Scholars have defined information overload in relation to the quantity and quality of the information and the cognitive responses toward the information (Eppler and Mengis, 2004; Ji et al., 2014). Accordingly, information overload consists of the following key components: (1) an overflow of information, (2) information characterized by ambiguity, and (3) ineffective management of information due to limited capacity (Kim et al., 2007). Based on these studies, we define perceived information overload as a state of feeling overwhelmed due to exposure to an excessive amount of complex, ambiguous, and uncertain COVID-19 information on WeChat and a limited capacity to process this information.

We employ the cognitive overload approach to illustrate the relationship between perceived information overload and unverified information sharing. The cognitive overload approach assumes that the human working memory has a limited capacity and that only a small amount of new information can be processed at a time (Sweller, 2011). When overloaded by complex and excessive messages, individuals tend to make careless decisions, such as accepting incoming messages without verification, as they experience less self-control and are unable to process these messages (Fox et al., 2007; Samson and Kostyszyn, 2015). Because perceived information overload is a major indicator of the cognitive overload approach (Whelan et al., 2020), we regard unverified information sharing as an outcome of perceived information overload.

Within the cognitive overload approach, the coping theory helps us further understand why people share information without verification when they experience information overload. The coping theory argues that individuals tend to make behavioral changes to manage psychological stress (Lazarus and Folkman, 1984; Tennen et al., 2000). Specifically, individuals employ the problem-focused coping strategy to solve the perceived problem by doing something to alter the source of stress (Lazarus and Folkman, 1984). Perceived information overload is a major source of psychological stress during the pandemic (Bermes, 2021). To alter this stressful encounter, individuals adjust their behaviors (Livneh and Martz, 2007). As a result, unverified information sharing, which requires little cognitive effort, represents a behavioral adaptation to manage the stressful state of information overload. Moreover, the positive association between perceived information overload and unverified information sharing has been empirically supported in previous studies (Talwar et al., 2019; Islam et al., 2020; Laato et al., 2020). Accordingly, we put forward the following hypothesis to examine unverified information sharing on WeChat:

*Hypothesis* 1 *(H1)*: Perceived information overload is positively associated with unverified information sharing.

### The Mediating Role of Anxiety

The COVID-19 pandemic has severely threatened people’s mental wellbeing and caused major emotional distress (Sheek-Hussein et al., 2021). Across the globe, high rates of anxiety have been reported in the general population during the pandemic (Xiong et al., 2020; Santabárbara et al., 2021). Anxiety is a future-oriented mood state that arises when individuals experience the risk of upcoming negative events (Freiling et al., 2021; Sampaio et al., 2021). The mutation of the coronavirus and the uncertainty about pandemic control globally denote a great risk to public wellbeing (Gomez et al., 2021). In the face of the risk, individuals tend to have a feeling of anxiety, a feeling of tension and worriedness, together with physical changes, such as increased blood pressure, sweating, trembling, dizziness, and a rapid heartbeat (Kazdin, 2000).

On the one hand, an individual’s anxiety about the pandemic can be exacerbated by his or her perceived information overload (Khaleel et al., 2020). The constant information influx on COVID-19 makes it difficult for people to differentiate between facts and rumors, which increases their stress in managing uncertainty (Mohammed et al., 2021). In such a circumstance, people’s perceived control over information seeking and processing decreases (Swar et al., 2017). An individual’s inability to access, understand, and make use of pertinent information might make this person anxious (Bawden and Robinson, 2009). Moreover, an abundance of studies have demonstrated that the overconsumption of COVID-19 information and the associated perceived information overload are positively correlated with anxiety (Holmes et al., 2020; Siebenhaar et al., 2020; Bendau et al., 2021; Song et al., 2021). Thus, we posit the following hypothesis:

*Hypothesis* 2 *(H2)*: Perceived information overload is positively associated with anxiety.

On the other hand, to cope with anxiety, people tend to engage in unverified information sharing. According to the coping theory, emotion-focused coping is aimed at managing or reducing the emotional distress caused by a given situation (Lazarus and Folkman, 1984). The severe and uncertain threat of the COVID-19 pandemic to public health has triggered anxiety among the general population (Xiong et al., 2020; Santabárbara et al., 2021). To manage anxiety and reduce emotional distress, people share information with their family, friends, co-workers, and community members to feel connected to close others (Chen et al., 2021; Lim et al., 2021). The positive association between anxiety and information sharing on social media has been empirically supported in previous research (Thelwall and Thelwall, 2020; Yin et al., 2020; Sharma and Kapoor, 2021). Because individuals in an anxious state are likely to make careless decisions during public health emergencies (Moghanibashi-Mansourieh, 2020), their information-sharing behaviors are often characterized by a lack of verification. Consequently, we expect that the more anxious an individual is about the pandemic, the more likely that he or she is to share information without authentication. More formally, we posit the following hypothesis:

*Hypothesis* 3 *(H3)*: Anxiety is positively associated with unverified information sharing.

The above postulated hypotheses suggest that anxiety may mediate the association between perceived information overload and unverified information sharing. Based on the stimulus-organism-response paradigm, a recent study demonstrated that external stimuli (e.g., perceived information overload) affected individuals’ internal states (e.g., anxiety) and their subsequent information behaviors amid the COVID-19 pandemic (Song et al., 2021). Because unverified information sharing is a specific type of information behavior, Song’s et al. (2021) findings provide a rationale for us to examine the following mediation effect:

*Hypothesis* 4 *(H4)*: Anxiety mediates the association between perceived information overload and unverified information sharing.

### The Moderating Role of Perceived Herd

Based on the social impact theory, individual behaviors are usually influenced by the presence of others’ actions (Latané, 1981; Handarkho, 2020). When individuals observe a large number of others performing a certain behavior, this person is also highly likely to perform the same behavior (Apuke and Omar, 2020). Thus, whether or not an individual shares a piece of information without authentication is susceptible to the number of others’ information-sharing behaviors observed on WeChat. We introduce perceived herd to illustrate this social impact on people’s unverified information sharing. Perceived herd refers to one’s willingness to follow a behavior performed by a substantial number of others (Handarkho, 2020). In this study, we define perceived herd as people’s willingness to share a piece of information when it is shared by a large number of others on WeChat. Specifically, we test whether perceived herd moderates the direct link between perceived information overload and unverified information sharing and the indirect link between them *via* anxiety.

The mechanism of herding behaviors helps explain the moderating role of perceived herd in the relationships between perceived information overload, anxiety, and unverified information sharing. Herding behaviors can be seen as imitating others and discounting one’s own decision (Sun, 2013). Herding behaviors usually occur under two conditions: uncertainty about the decision and observation of others’ actions (Sun, 2013). In other words, when an individual feels uncertain about whether or not to perform a certain behavior, that person is likely to imitate others. Notably, the likelihood of imitating others increases if an individual observes that a considerable number of others are performing the behavior. For instance, during the early COVID-19 outbreak, many people were uncertain about whether to stock up; nevertheless, when they noticed that a majority of others were engaging in panic buying, they followed such a behavior (Loxton et al., 2020). Likewise, when individual users are uncertain about whether to share a social media post or endorse an online review, they tend to imitate others; if they observe many “likes” of the post and many favorable online reviews, they will follow the crowd and perform the same behavior (Mattke et al., 2020; Xue et al., 2020). These studies demonstrate that perceived herd may largely increase an individual’s likelihood of performing a behavior about which he or she is previously uncertain.

People’s perceived information overload and the associated anxiety are often accompanied by their uncertainty about the veracity of information related to COVID-19 (Mohammed et al., 2021). Thus, people may hesitate to share this uncertain information. However, their hesitation may decrease when they observe a considerable number of close others and influential users have shared uncertain information on social media. In such circumstances, people are more likely to herd and share the same information, usually without verification (Rao et al., 2001; Apuke and Omar, 2020). Thus, we propose that perceived herd may moderate the link between perceived information overload and unverified information sharing and the link between anxiety and unverified information sharing. For individuals with high levels of perceived herd, the association between perceived information overload and unverified information sharing will be stronger compared with those with low levels of perceived herd. Similarly, the association between anxiety and unverified information sharing will be stronger for individuals with high levels of perceived herd than for those with low levels of perceived herd. We put forward the following hypotheses to test the moderating role of perceived herd:

*Hypothesis* 5 *(H5)*: Perceived herd positively moderates the direct link between perceived information overload and unverified information sharing. The direct link between perceived information overload and unverified information sharing is stronger in conditions where the level of perceived herd is high than in conditions where the level of perceived herd is low.

*Hypothesis* 6 *(H6)*: Perceived herd positively moderates the link between anxiety and unverified information sharing. The link between anxiety and unverified information sharing is stronger in conditions where the level of perceived herd is high than in conditions where the level of perceived herd is low.

Moreover, considering that perceived herd moderates the association between anxiety and unverified information sharing, perceived herd is also likely to moderate the indirect effect. Thus, we posit another hypothesis:

*Hypothesis* 7 *(H7)*: Perceived herd positively moderates the indirect effect of perceived information overload on unverified information sharing *via* anxiety. The indirect effect of perceived information overload on unverified information sharing *via* anxiety is stronger in conditions where the level of perceived herd is high than in conditions where the level of perceived herd is low.

**Figure 1:**
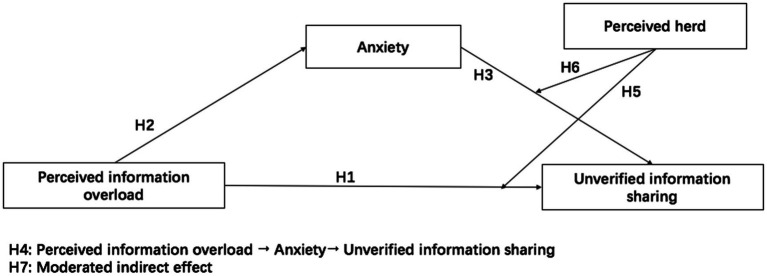
Hypothesized model.

Figure 1 presents the hypothesized model in this study.

## Materials and Methods

### Participants

A cross-sectional online survey was conducted to collect the data. We recruited participants by using Sojump’s sampling service,^1^ a professional online survey website in China. The sampling pool provided by Sojump consists of 2.6 million registered respondents with diverse demographic characteristics in mainland China. A number of previous studies have used this sampling strategy to examine various social issues in China, such as air pollution, renewable energy use, and the development of e-commerce (Zhou et al., 2013; Chen et al., 2016; Huang, 2020). Our survey began on December 6, 2021, and ended on December 8, 2021. By December 8, 2021, the COVID-19 pandemic was generally under control in China, but small-scale outbreaks were occurring in local areas: eight high-risk areas and 44 medium-risk areas were noted across the country (The State Council of the People's Republic of China, 2021). A plethora of information related to COVID-19 circulated on WeChat during this time period. Through exposure to this information, individuals may experience information overload and anxiety. Furthermore, they could observe others’ information-sharing behaviors on WeChat. Thus, during this time period, respondents might feel information overload, experience anxiety and perceived herd, and engage in unverified information sharing on WeChat, although the degree of these variables might differ between respondents. The institutional review board of the authors’ university approved the data collection protocol. Voluntary informed consent was obtained from the participants before the online survey.

To be eligible for this study, participants had to have experience using WeChat to acquire the COVID-19 information. A total of 556 participants in Sojump’s survey pool completed the online survey. We considered questionnaires invalid if they met one of the two criteria: (1) made multiple submissions using the same IP address or (2) did not pass any of the five attention checks (e.g., “please select ‘strongly agree’”). Finally, 525 valid cases were used for the data analysis. Table 1 displays the demographic features of the participants.

**Table 1:** Demographic characteristics of the participants.

Measure	Item	Frequency	Percentage (%)
Gender	Male	217	41.3
	Female	308	58.7
Age	18–24	39	7.4
	25–34	279	53.1
	35–44	98	18.7
	45–65	106	20.2
	Over 65	3	0.6
Education level	Never attend to school	0	0
	Primary school	0	0
	Middle school	5	1.0
	High school	17	3.2
	Vocational high school	13	2.5
	Higher vocational school	64	12.2
	Bachelor	383	73.0
	Master	41	7.8
	PhD	2	0.4
Monthly income	Less than 1,500 RMB	7	1.3
	1,501–2,000 RMB	6	1.1
	2,001–3,000 RMB	13	2.5
	3,001–5,000 RMB	74	14.1
	5,001–8,000 RMB	161	30.7
	8,001–12,000 RMB	140	26.7
	12,001–20,000 RMB	96	18.3
	More than 20,000 RMB	28	5.3

### Measures

#### Perceived Information Overload

Referring to prior research (Laato et al., 2020), perceived information overload was measured with three items on a 5-point Likert scale (1 = “strongly disagree,” 5 = “strongly agree”): (1) “I am often distracted by the excessive amount of the COVID-19 information on WeChat,” (2) “I find that I am overwhelmed by the amount of the COVID-19 information on WeChat that I process on a daily basis,” and (3) “I receive too much information regarding the COVID-19 pandemic to form a coherent picture of what’s happening.” The three items were averaged, with higher scores suggesting higher levels of perceived information overload (*M* = 2.89, *SD* = 0.92, Cronbach’s *α* = 0.76).

#### Anxiety

The measurement of anxiety was developed through adapting two previous scales (Lovibond and Lovibond, 1995; He et al., 2019). Participants were asked to indicate the extent to which they experienced the following feelings about the COVID-19 pandemic: (1) anxious, (2) panicky, (3) terrified, (4) scared, and (5) dizzy. The items were measured on a 7-point scale (1 = “not at all,” 7 = “very strongly”). The five items were averaged to create a composite index, with higher values indicating higher levels of anxiety (*M* = 3.45, *SD* = 1.23, Cronbach’s *α* = 0.88).

#### Perceived Herd

In accordance with a previous instrument (Apuke and Omar, 2020), we measured perceived herd with three items on a 5-point Likert scale (1 = “strongly disagree,” 5 = “strongly agree”): (1) “My choice to share the COVID-19 information on WeChat is influenced by the number of people who like and share it,” (2) “If I realized that many of my friends share certain COVID-19 information on WeChat, then I would be more willing to share this information,” and (3) “The more people like and share the COVID-19 information on WeChat, the more likely it is for me to reshare it.” A composite index was created by calculating the mean score of the three items, with a higher value indicating a higher degree of perceived herd (*M* = 3.22, *SD* = 0.96, Cronbach’s *α* = 0.84).

#### Unverified Information Sharing

Following a previous instrument (Laato et al., 2020), we used four items to measure the frequency of unverified information sharing on WeChat: (1) “How often do you share information or news related to COVID-19 on WeChat without checking its authenticity?,” (2) “How often do you share information or news about COVID-19 on WeChat without checking facts through trusted sources?,” (3) “How often do you share information or news related to COVID-19 on WeChat without verifying it?,” and (4) “How often do you share information or news related to COVID-19 on WeChat even if sometimes you feel the information may not be correct?.” Participants answered the questions on a 5-point Likert scale (1 = “never,” 5 = “always”). The four items were averaged to create an additive index of unverified information sharing (*M* = 1.61, *SD* = 0.76, Cronbach’s *α* = 0.87).

#### Control Variables

Age was measured as a continuous variable (*M* = 35.14, *SD* = 9.83) and gender as a dichotomous variable (41.3% males). Monthly income (Median = 6.00, or 8,001–12,000 RMB/month, *SD* = 1.33) and education level (Median = 7.00, or Bachelor’s degree, *SD* = 0.84) were both measured as ordinal variables. In addition, considering that exposure frequency was associated with information sharing (He et al., 2019), we included it as a control variable. A single item was used to measure exposure frequency on a 5-point scale (1 = “never,” 5 = “always”): “How often do you encounter information or news related to COVID-19 in the past month?” (*M* = 3.50, *SD* = 0.82).

### Statistical Analyses

We first used SPSS version 26.0 to calculate the means and standard deviations of the examined variables and the bivariate correlations between them. Then, we employed PROCESS version 3.5 to test the research hypotheses. Age, gender, monthly income, education level, and WeChat exposure frequency were entered as covariates in the analysis. The mediating role of anxiety between perceived information overload and unverified information sharing was tested using Model 4 of the PROCESS macro (Hayes, 2013). The moderating role of perceived herd in the mediation model was tested using Model 15 of the PROCESS macro (Hayes, 2013). We tested the mediation effect and moderated mediation effect with 5,000 bootstrap samples at 95% bias-corrected confidence intervals (Preacher and Hayes, 2008). A bootstrap confidence interval that did not include zero indicated a significant effect. Unstandardized coefficients were reported.

## Result

### Preliminary Analyses

Table 2 presents a correlation matrix of the variables. Perceived information overload was positively correlated with unverified information sharing (*r* = 0.28, *p* < 0.001) and anxiety (*r* = 0.41, *p* < 0.001). Both anxiety (*r* = 0.29, *p* < 0.001) and perceived herd (*r* = 0.42, *p* < 0.001) were positively associated with unverified information sharing. Among the control variables, exposure frequency was positively correlated with unverified information sharing (*r* = 0.18, *p* < 0.001), while age was negatively correlated with unverified information sharing (*r* = −0.14, *p* < 0.01).

**Table 2:** Correlations between the variables.

	UIS	PIO	Anxiety	PH	Exposure	Gender	Age	Education	Income
UIS	1								
PIO	0.28^***^	1							
Anxiety	0.29^***^	0.41^***^	1						
PH	0.42^***^	0.25^***^	0.21^***^	1					
Exposure	0.18^***^	0.17^***^	0.13^**^	0.22^***^	1				
Gender	− 0.05	− 0.002	0.06	− 0.02	− 0.04	1			
Age	− 0.14^**^	− 0.11^*^	− 0.18^***^	− 0.07	− 0.04	− 0.28^***^	1		
Education	0.06	0.01	0.04	− 0.03	0.08	0.07	− 0.35^***^	1	
Income	− 0.02	− 0.08	0.02	0.01	0.07	− 0.07	0.01	0.33^***^	1

*** *p* < 0.001;

** *p* < 0.01; and

* *p* < 0.05.

*N* = 525. UIS, unverified information sharing; PIO, perceived information overload; and PH, perceived herd.

### The Mediating Role of Anxiety

To test the mediating role of anxiety in the relationship between perceived information overload and unverified information sharing, a mediation analysis was performed. Exposure frequency, gender, age, education level, and monthly income were entered as covariates. Perceived information overload was entered as the independent variable, unverified information sharing as the outcome variable, and anxiety as the mediator variable. The statistical results are shown in Table 3.

**Table 3:** Testing the mediating role of anxiety.

Predictors	Model 1	Model 2	Model 3
	UIS	Anxiety	UIS
	*B* (*SE*) *t*	*B* (*SE*) *t*	*B* (*SE*) *t*
PIO	0.20 (0.04) 5.70^***^	0.52 (0.05) 9.56^***^	0.14 (0.04) 3.69^***^
Anxiety			0.12 (0.03) 4.28^***^
*R* ^2^	0.11	0.19	0.14
*F*	10.70^***^	20.62^***^	12.09^***^

*** *p* < 0.001.

*N* = 525. Each column is a regression model which predicts the criterion at the top of the column. Unstandardized coefficients were reported. UIS, unverified information sharing; PIO, perceived information overload.

Supporting H1, a positive association was found between perceived information overload and unverified information sharing (*B* = 0.14, *SE* = 0.04, *p* < 0.001). Consistent with H2 and H3, perceived information overload was positively associated with anxiety (*B* = 0.52, *SE* = 0.05, *p* < 0.001), and anxiety was positively correlated with unverified information sharing (*B* = 0.12, *SE* = 0.03, *p* < 0.001). In addition, the bootstrap analysis demonstrated that the indirect effect of perceived information overload on unverified information sharing *via* anxiety was significant (effect size = 0.06, *SE* = 0.02, CI [0.03, 0.10]). Thus, H4 was supported.

### Moderated Mediation Effect

To test H5–H7, we examined the moderated mediation effect. Exposure frequency, gender, age, education level, and monthly income were treated as covariates. Perceived information overload was entered as the independent variable, unverified information sharing as the outcome variable, anxiety as the mediator variable, and perceived herd as the moderator variable. Three conditions were created based on the value of the moderator variable (Hayes, 2013): one standard deviation below the mean (2.00), the mean (3.33), and one standard deviation above the mean (4.33).

**Figure 2:**
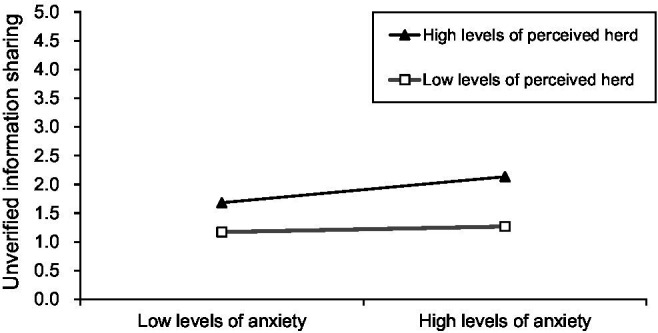
Interaction effect of anxiety and perceived herd on unverified information sharing.

**Figure 3:**
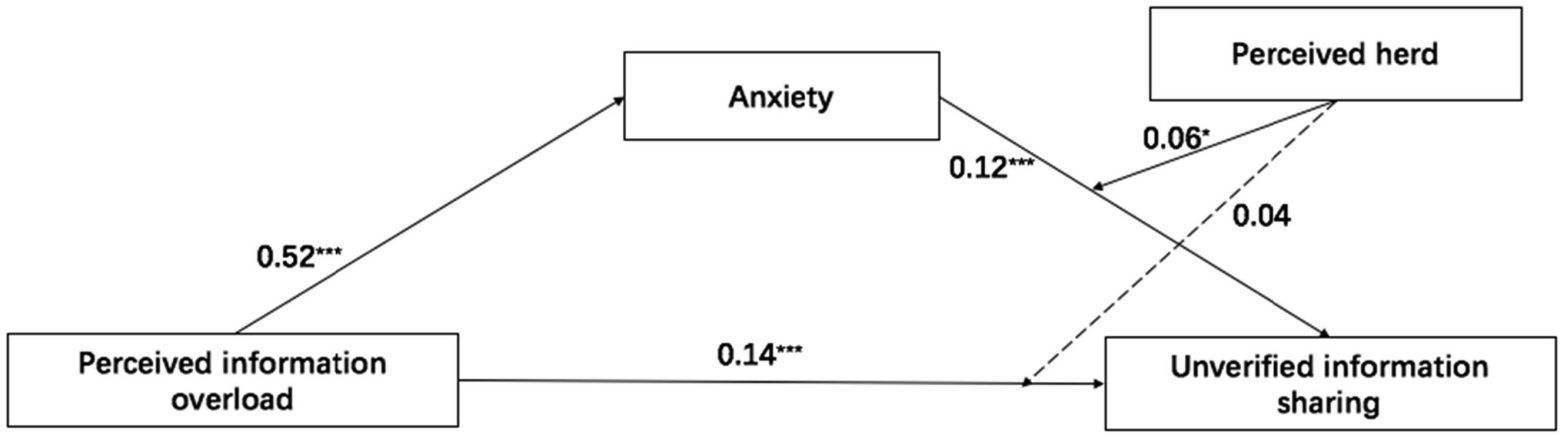
Final model based on statistical results. ^*^p < 0.05 and ^***^p < 0.001.

Inconsistent with H5, the results showed that the interaction effect of perceived information overload and perceived herd on unverified information sharing was not significant (*B* = 0.04, *SE* = 0.04, *p* = 0.23). Supporting H6, we found that the interaction effect of anxiety and perceived herd on unverified information sharing was significant (*B* = 0.06, *SE* = 0.03, *p* < 0.05). The significant interaction effect was further examined using simple slope analysis. We plotted the interaction effect of anxiety and perceived herd on unverified information sharing in Figure 2. Notably, the association between anxiety and unverified information sharing was stronger for people with high levels of perceived herd (simple slope = 0.16, *t* = 4.11, *p* < 0.001) than for those with low levels of perceived herd (simple slope = 0.10, *t* = 3.83, *p* < 0.001). Moreover, perceived herd moderated the indirect effect of perceived information overload on unverified information sharing *via* anxiety: the indirect effect was significant in conditions where the level of perceived herd was high (effect size = 0.08, *SE* = 0.03, CI [0.03, 0.14]), whereas the indirect effect was not significant in conditions where the level of perceived herd was low (effect size = 0.01, *SE* = 0.01, CI [−0.0, 0.04]). Hence, H7 was supported. Table 4 presents the results of the moderated mediation analysis. Figure 3 depicts the final model based on the statistical results.

**Table 4:** Testing the moderated mediation effect.

Predictors	Model 1	Model 2
	Anxiety	UIS
	*B* (*SE*) *t*	*B* (*SE*) *t*
PIO	0.52 (0.05) 9.56^***^	−0.06 (0.12) −0.52
PIO × PH		0.04 (0.04) 1.21
Anxiety		−0.10 (0.08) −1.22
Anxiety × PH		0.06 (0.03) 2.44^*^
*R* ^2^	0.19	0.26
*F*	20.62^***^	18.45^***^

* *p* < 0.05 and

*** *p* < 0.001.

*N* = 525. Unstandardized coefficients were reported. UIS, unverified information sharing; PIO, perceived information overload.

## Discussion

Although a number of studies have demonstrated the association between perceived information overload and unverified information sharing (Talwar et al., 2019; Islam et al., 2020; Laato et al., 2020), the potential mechanisms underlying the process remain underexplored. To this end, the present study proposes a moderated mediation model to test the mediating role of anxiety and the moderating role of perceived herd. The results showed a direct and positive association between perceived information overload and unverified information sharing. Furthermore, the mediating role of anxiety demonstrated that as perceived information overload increased, anxiety intensified, which then facilitated the behavior of unverified information sharing. Moreover, perceived herd moderated this mediating effect: the indirect effect of perceived information overload on unverified information sharing *via* anxiety was significant in conditions where the level of perceived herd was high, whereas the indirect effect was not significant in conditions where the level of perceived herd was low.

### Theoretical Implications

First, consistent with our hypothesis, this study showed that perceived information overload facilitated unverified information sharing on WeChat. The finding supported that individuals overloaded by large packets of complex information had limited processing capability and tended to make quick decisions without a second thought (Sweller, 2011; Samson and Kostyszyn, 2015). Meanwhile, this quickly made decision—unverified information sharing in the current study—served as a coping strategy for individuals to resolve the problem of perceived information overload (Lazarus and Folkman, 1984). Thus, our findings corroborated the cognitive overload approach (Fox et al., 2007; Talwar et al., 2019; Laato et al., 2020) in explicating people’s unverified information sharing on social media, especially in circumstances characterized by an imminent threat and high levels of uncertainty, such as the COVID-19 pandemic.

Second, our results showed that anxiety partially mediated the association between perceived information overload and unverified information sharing. On the one hand, the mediation effect exhibited that perceived information overload induced anxiety. This finding may help researchers better understand the antecedents of anxiety in information-sharing research, which has rarely been examined. On the other hand, the mediation effect showed that anxiety triggered unverified information sharing. Compared with previous research that identified anxiety as a predictor of information sharing (Thelwall and Thelwall, 2020; Yin et al., 2020; Sharma and Kapoor, 2021), our findings demonstrated that anxiety also predicted unverified information sharing. Moreover, the findings also supported that unverified information sharing functioned as a coping strategy for individuals to deal with emotional distress (Lazarus and Folkman, 1984) during the pandemic. Taken together, this mediation effect extended the cognitive overload approach by including anxiety as an emotional outcome of information overload and an emotional predictor of unverified information sharing.

Third, the moderated mediation effect demonstrated that the indirect effect of perceived information overload on unverified information sharing *via* anxiety was significant only in conditions where the level of perceived herd was high, whereas the indirect effect was not significant in conditions where the level of perceived herd was low. As previously discussed, the mediation effect suggested that unverified information sharing served as a strategy for individuals to cope with the anxiety induced by information overload. However, the moderated mediation effect indicated that this coping mechanism worked only when individuals had high levels of perceived herd. Consistent with the social impact theory (Latané, 1981; Apuke and Omar, 2020; Handarkho, 2020), these results suggested that people’s unverified information sharing on social media was susceptible to the influence of others’ information sharing behaviors. Moreover, whether individuals engaged in unverified information sharing to cope with anxiety largely depended on their levels of perceived herd. Compared with prior research that focused on the cognitive and emotional predictors of unverified information sharing (He et al., 2019; Talwar et al., 2019; Islam et al., 2020; Laato et al., 2020; Lim et al., 2021), the moderating role of perceived herd highlighted that social influence might precondition cognitive and emotional effects on one’s unverified information sharing.

Fourth, inconsistent with our hypothesis, we found that perceived herd did not significantly moderate the direct association between perceived information overload and unverified information sharing. This is probably because behaviors driven by emotions are highly susceptible to perceived herd, whereas perceived herd makes little difference to the occurrence of behaviors based on cognitive judgment (Loxton et al., 2020). In the current study, the direct association between perceived information overload and unverified information sharing represented a process of how one’s cognition of information overload triggered their behavior of unverified information sharing. Thus, this direct association was not easily affected by perceived herd.

### Practical Implications

The moderated mediation model proposed in this study has some practical implications for managing people’s unverified information sharing on WeChat and other social media platforms. First, the direct association between perceived information overload and unverified information sharing suggests that individuals’ perceived information overload should be decreased to lower their tendency to engage in unverified information sharing. This can be realized in several ways. Social media platforms should improve gatekeeping functions, such as setting up effective fact checkers to filter out a vast number of misinformation. Meanwhile, platforms can use algorithms to push information on other topics to divert users’ attention to COVID-19 information. Furthermore, based on users’ web browsing records, platform corporations can target heavy users who are likely to experience information overload and set a protective mode to prevent them from browsing posts related to the coronavirus for too long. Social media users are encouraged to enhance their digital literacy so that they are more capable of dealing with the information influx and thus reduce the feeling of perceived information overload.

Second, given that anxiety mediated the relationship between perceived information overload and unverified information sharing, anxiety should be regulated to properly manage unverified information sharing on social media. For instance, social media platforms are advised to insert a note in sections of COVID-19-related information, thereby reminding users to stay alert to the content that may trigger their anxiety and other negative feelings. Furthermore, AI-powered chatbots can be built in the browsing interface for users to initiate a conversation if necessary, thus easing users’ anxiety caused by information overload. In addition to these online measures, users are advised to regulate their anxiety by themselves, such as seeking emotional support from close ones or reappraising the encounter of information overload.

Third, the indirect effect of perceived information overload on unverified information *via* anxiety was only significant in conditions where the level of perceived herd was high, which indicates that special attention should be paid to social media users with high levels of perceived herd. Through lawfully analyzing users’ digital footprints on social media, platforms can target groups of users who often herd in terms of information sharing. To reduce the perceived herd of these targeted groups, platforms can use algorithms to recommend diverse topics and views to them.

### Limitations and Future Research

The current study has several limitations. First, we collected data only from China. Because unverified information sharing related to the COVID-19 pandemic on social media has become a common problem in many countries across the globe (Islam et al., 2020; Laato et al., 2020; Apuke and Omar, 2021), the one country- and single platform-based design provides us with limited insights into understanding this problem. Thus, in the future, scholars can conduct comparative research to examine unverified information sharing on different social media platforms between different countries. Furthermore, we could not claim causality between the examined variables, as we used a cross-sectional design. Hence, experimental or longitudinal studies can be used to test causal relationships in the future. In addition, the mean value of unverified information sharing on WeChat was low. This is probably because our study was conducted during a time period when the COVID-19 pandemic was not very salient in China. Accordingly, future research can test the moderated mediation model in areas where the pandemic is salient. Lastly, the self-reported measures of unverified information sharing in this study, which were also widely used in prior research (Islam et al., 2020; Laato et al., 2020), were prone to social desirability and estimation biases. To overcome this limitation, we advise researchers to use an experimental design to observe subjects’ unverified information sharing.

## Conclusion

This study proposes a moderated mediation model to unveil the socio-psychological mechanism of people’s unverified information sharing on WeChat during the COVID-19 pandemic. Perceived information overload predicts unverified information sharing. Furthermore, this relationship is partially mediated by anxiety. Moreover, the indirect path between perceived information overload and unverified information sharing through anxiety is significant only in conditions where the level of perceived herd is high. The findings indicate that unverified information sharing on social media not only serves as an individual strategy to cope with information overload but also represents a herding behavior to resolve anxiety. In relation to extant research on unverified information sharing, this moderated mediation model not only extends the cognitive overload approach by including anxiety as a mediator but also highlights that perceived herd may precondition the effects of cognitive and emotional predictors on unverified information sharing. The model also provides social media platforms and social media users with some practical implications to lower the tendency toward unverified information sharing. Hopefully, our study could offer some insights into curbing the widespread misinformation and fighting the infodemic.

## Data Availability Statement

The raw data supporting the conclusions of this article will be made available by the authors, without undue reservation.

## Ethics Statement

The studies involving human participants were reviewed and approved by the Institutional Review Board of the Faculty of Social Sciences, Zhejiang University. The patients/participants provided their written informed consent to participate in this study.

## Author Contributions

QH acquired funding, designed the study, analyzed the data, and wrote the main body of the manuscript. SL participated in the study design, cleaned the data, wrote the “Result” section of the manuscript, and made all the tables and figures. BN participated in the study design and data analysis and wrote the “Materials and Methods” section of the manuscript. All authors contributed to the article and approved the submitted version.

## Funding

This study was supported by the National Social Science Foundation of China (19CXW029).

## Conflict of Interest

The authors declare that the research was conducted in the absence of any commercial or financial relationships that could be construed as a potential conflict of interest.

## Publisher’s Note

All claims expressed in this article are solely those of the authors and do not necessarily represent those of their affiliated organizations, or those of the publisher, the editors and the reviewers. Any product that may be evaluated in this article, or claim that may be made by its manufacturer, is not guaranteed or endorsed by the publisher.

## Footnotes

^1^ http://www.sojump.com
